# GLUT4, GLUT1, and GLUT8 are the dominant GLUT transcripts expressed in the murine left ventricle

**DOI:** 10.1186/1475-2840-11-63

**Published:** 2012-06-08

**Authors:** Lauren Aerni-Flessner, Melissa Abi-Jaoude, Amanda Koenig, Maria Payne, Paul W Hruz

**Affiliations:** 1 Department of Pediatrics, Washington University School of Medicine, St. Louis, MO, USA; 2 Department of Cell Biology and Physiology, Washington University School of Medicine, St. Louis, MO, USA

**Keywords:** Cardiomyopathy, Diabetes, Gene expression, Glucose transport, Heart failure

## Abstract

**Background:**

The heart derives energy from a wide variety of substrates including fatty acids, carbohydrates, ketones, and amino acids. The healthy heart generates up to 30% of its ATP from glucose. Under conditions of cardiac injury or stress, the heart relies even more heavily on glucose as a source of fuel. Glucose is transported into the heart by members of the family of facilitative glucose transporters (GLUTs). While research examining the transport of glucose into the heart has primarily focused on the roles of the classical glucose transporters GLUT1 and GLUT4, little is known about the functions of more newly identified GLUT isoforms in the myocardium.

**Methods:**

In this study the presence and relative RNA message abundance of each of the known GLUT isoforms was determined in left ventricular tissue from two commonly used inbred laboratory mouse strains (C57BL/6J and FVB/NJ) by quantitative real time PCR. Relative message abundance was also determined in GLUT4 null mice and in murine models of dilated and hypertrophic cardiomyopathy.

**Results:**

GLUT4, GLUT1, and GLUT8 were found to be the most abundant GLUT transcripts in the normal heart, while GLUT3, GLUT10, and GLUT12 are present at relatively lower levels. Assessment of relative GLUT expression in left ventricular myocardium from mice with dilated cardiomyopathy revealed increased expression of GLUT1 with reduced levels of GLUT4, GLUT8, and GLUT12. Compensatory increase in the expression of GLUT12 was observed in genetically altered mice lacking GLUT4.

**Conclusions:**

Glucose transporter expression varies significantly among murine models of cardiac dysfunction and involves several of the class III GLUT isoforms. Understanding how these more newly identified GLUT isoforms contribute to regulating myocardial glucose transport will enhance our comprehension of the normal physiology and pathophysiology of the heart.

## Introduction

The mammalian heart demands a constant supply of ATP in order to perform its vital role in delivering oxygen, metabolic substrates, and hormones to peripheral tissues. In order to generate an adequate supply of ATP to meet this high energy demand, the heart requires a continuous supply of metabolic fuel. The healthy heart derives its energy from a wide variety of carbon based substrates including fatty acids, carbohydrates, ketones, and amino acids [1]. The flexibility that the healthy heart displays in its metabolic machinery enables it to alter substrate use depending on substrate availability and circulating hormone levels. While the healthy heart derives a majority of its energy from fatty acids, up to 30% of myocardial ATP is derived from glucose and lactate [2]. Furthermore, under conditions of myocardial injury or stress, such as during ischemia or during pressure overload hypertrophy, the heart displays reduced substrate flexibility with an increased dependence on glucose [3]. The transport of glucose into the myocardium is mediated by members of the facilitative glucose transporter (GLUT) family.

There are fourteen known members of the GLUT family: the Class I transporters GLUTs-1, -2, -3, -4, and −14, the Class II transporters GLUTs-5, -7, -9, and −11, and the Class III transporters GLUTs-6, -8, -10, -12, and HMIT [4,5]. These GLUT isoforms differ in their substrate specificity, kinetics of transport, and tissue distribution and localization. Many of the Class II and Class III isoforms in the GLUT family have been discovered only in recent years, and the specific role that the newly identified GLUTs play in mediating the transport of hexoses across the membranes of mammalian cells remains poorly understood.

The Class I transporters GLUT1 and GLUT4 are the most extensively studied GLUTs in mammalian tissues. GLUT4, the canonical insulin-responsive glucose transporter, is the predominant GLUT expressed in the adult heart [6]. GLUT1 is a major GLUT transporter expressed in the fetal heart [7]. GLUT1 also increases in abundance in the adult heart in response to myocardial injury or stress [8,9]. In addition to GLUTs-1 and −4, several of the newly identified GLUT transporters, including the Class III isoforms GLUTs-8, -10, and −12, have been detected in cardiac tissue [10-14]. These isoforms may represent additional insulin-responsive GLUTs, as GLUT8 has been identified as an insulin-responsive GLUT in blastocysts [15] and GLUT12 overexpression in mice has been shown to improve whole body insulin-sensitivity [16]. However, the functional role of these transporters in regulating myocardial glucose uptake remains largely unexplored. In addition, little quantitative data in regards to the expression or function of these GLUTs in the myocardium are available. To better understand the role for these newly identified GLUTs in the heart, we set out to identify which isoforms of the facilitative glucose transporter family are present in the left ventricle of mice. Furthermore, we have used real time PCR to determine quantitatively which GLUTs transcripts are most highly abundant in the heart. Identifying the GLUT isoforms that are most highly expressed in the heart under healthy settings, and determining how the expression levels of these GLUTs change under pathological circumstances provides novel insight into the potential functional roles of these newly identified GLUTs in the normal physiology and pathophysiology of the heart.

## Materials and methods

### Animal care

All mouse studies were approved by the Animal Studies Committee at Washington University School of Medicine and conform to the Guide for the Care and Use of Laboratory Animals published by the National Institute of Health. Mice were housed in the animal facility at Washington University under standard light/dark cycles and fed standard mouse chow diet and water *ad libitum*. Ten-week-old male FVB/NJ mice and C57BL/6J mice (The Jackson Laboratory) were used for the identification and quantification of GLUT transcripts in the left ventricle myocardium. Six-month-old male GLUT4 null mice and age matched C57BL/6J controls were used to identify the relative GLUT transcript levels in the murine left ventricle following genetic ablation of GLUT4. Twelve-week-old male TG9 mice and wild type (FVB/NJ) littermate controls were used to determine the relative abundance of GLUT transcripts in the left ventricle in a genetic model of dilated cardiomyopathy. Male C57BL/6J mice were used in the aortic banding studies described below in order to examine the relative abundance of GLUTs in the left ventricle during pathophysiological hypertrophy.

### Transverse aortic constriction

Ten-week-old C57BL/6J male mice were randomly assigned to either a sham-operated or a transverse aortic constriction (TAC) group. TAC was performed as described previously [17]. Sham-operated mice endured the same surgical procedure but the aortic constriction was not placed. After seven days, surviving animals were sacrificed and the left ventricles were dissected out and weighed. The left ventricles were then frozen and RNA was isolated as described below.

### RNA isolation and real-time PCR

Total RNA was isolated using the Trizol® Plus RNA Purification System (Invitrogen, Carlsbad, CA), and one microgram of RNA was reverse transcribed using the SuperScript® III First-Strand Synthesis System for RT-PCR (Invitrogen, Carlsbad, CA). PCR was performed using Klentaq1 (DNA Polymerase Technology, St. Louis, MO) to identify the GLUT RNA transcripts that are present in the left ventricles of male FVB/NJ and C57BL/6J mice. Quantitative RT-PCR was performed using Power SYBR® Green PCR Master Mix (Applied Biosystems, Foster City, CA). Each reaction was run in triplicate using the validated primers listed in Table 1. Quantifications were performed with standard curves generated using pCR 2.1-TOPO plasmids (Invitrogen, Carlsbad, CA) that contain each GLUT cDNA amplicon. To control for the intrinsic variability in the efficiency of the reverse transcription reactions for the RNA samples used in this study, the results were normalized to β-*Actin* as described previously [18]. Briefly, the cycle threshold (Ct) values for β-*Actin* that were obtained from running individual cDNA samples in triplicate were averaged. The average β-*Actin* Ct values for the entire pool of left ventricle samples was then determined and this value was used as a baseline with the ΔΔCt method in order to create a normalization factor for each individual left ventricle sample. The GLUT copy numbers that were generated using the standard curve described above for each left ventricle sample were then normalized to the value obtained using β-*Actin* as a normalization factor. Since β-*Actin* transcript levels can change in heart failure [19], the relative changes in GLUT expression in murine models of cardiac hypertrophy compared to controls animals were determined using the ΔΔCt method with HPRT serving as an internal control.

**Table 1:** Murine glucose transporter primers

	**Forward primer**	**Reverse primer**	**Amplicon(bp)**
Glut1	TCAACACGGCCTTCACTG	CACGATGCTCAGATAGGACATC	164
Glut2	TGTGCTGCTGGATAAATTCGCCTG	AACCATGAACCAAGGGATTGGACC	109
Glut3	TTCTGGTCGGAATGCTCTTC	AATGTCCTCGAAAGTCCTGC	143
Glut4	GTAACTTCATTGTCGGCATGG	AGCTGAGATCTGGTCAAACG	155
Glut5	GGCTCATCTTCCCCTTCATTC	ATGAATGTCCTGCCCTTGG	145
Glut6	TTGGTGCTGTGAGGCT	TGGCACAAACTGGACGTA	140
Glut8	TTCATGGCCTTTCTAGTGACC	GAGTCCTGCCTTTAGTCTCAG	145
Glut9	TGCTTCCTCGTCTTCGCCACAATA	CTCTTGGCAAATGCCTGGCTGATT	113
Glut10	ACCAAAGGACAGTCTTTAGCTG	ATCTTCCAAGCAGACGGATG	148
Glut12	GGGTGTCAACCTTCTCATCTC	CCAAAGAGCATCCCTTAGTCTC	149
Actin	GATTACTGCTCTGGCTCCTAG	GACTCATCGTACTCCTGCTTG	147
HPRT	CCCCAAAATGGTTAAGGTTGC	AACAAAGTCTGGCCTGTATCC	76

### Western blotting

Left ventricular myocardium was harvested from mice immediately following euthanasia and was flash frozen in liquid nitrogen. Lysates were prepared by homogenizing the frozen ventricles in lysis buffer containing 1% triton X100, 1 mM sodium vanadate, 50 mM sodium fluoride, 10 mM sodium pyrophosphate, and protease inhibitor cocktail (Sigma, Saint Louis, MO) in PBS. Lysates were incubated on ice for 30 minutes and cleared by centrifugation at 1500xg for 10 minutes at 4°C. Protein concentration was determined using the Pierce BCA Protein Assay Kit (Pierce biotechnology, Rockford, IL). The GLUT8 and GLUT12 antibodies were a kind gift from Dr. Kelle Moley. Western blot analysis was then performed using 10 μg of total protein per lane. GLUT1, GLUT4, and GLUT12 antibodies recognizing the COOH-terminus of the transporters were used at a 1:1000 dilution in 5% milk in TBS-T. A GLUT8 antibody recognizing the NH_2_-terminus of the transporter was used at a 1:1000 dilution in 5% milk in TBS-T. Monoclonal GAPDH (Sigma, Saint Louis, MO) was used as an internal control for loading variability. Blots were imaged and relative protein levels were determined using the Odyssey Infrared Imaging System Version 3.0 (LI-COR Biosciences, Lincoln, NE).

### Statistical analyses

The data are reported as means ± SEM. Differences between control and experimental values were determined by Student’s *t* test. Statistical significance is set as P < 0.05.

## Results

### GLUT expression in left ventricle

**Figure 1:**
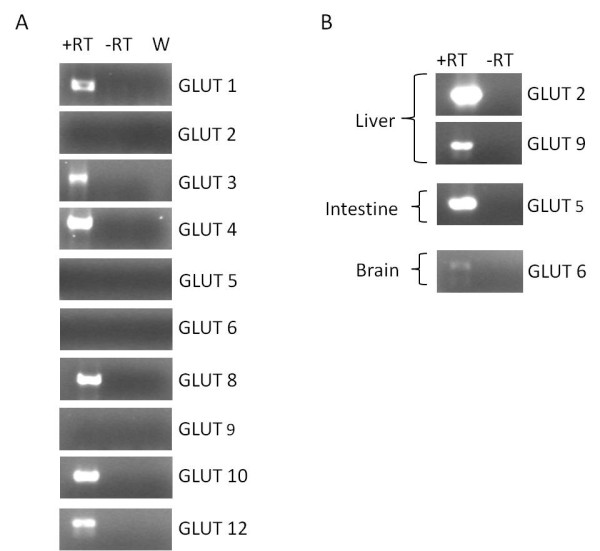
**Detection of GLUT mRNAs in the left ventricle of C57BL/6J mice. A**) PCR was employed to identify each known murine member of the facilitative glucose transporter family in the left ventricle of 10-week-old male C57BL/6J mice. GLUTs-1, -3, -4, -8, -10, and −12 were amplified using cDNA derived from the left ventricular myocardium. The identity of each GLUT amplicon was confirmed by sequencing. GLUTs-2, -5, -6, and −9 were not present in the left ventricle of male C57BL/6J mice. Representative images are shown (n = 3). **B**) The specificity of the primers used to amplify GLUTs-2, -5, -6, and −9 were confirmed using cDNA derived from the positive control tissues: liver, intestine, and brain. The specificity of the primers for each GLUT amplicon was confirmed by sequencing.

To determine the relative expression of glucose transporters in healthy hearts, two inbred strains of mice that are most commonly used in laboratory investigation of cardiac function (C57BL/6J and FVB/NJ) were selected for initial characterization of cardiac GLUT expression. Analysis of RNA obtained from left ventricle myocardium of ten-week-old male mice from both strains identified transcripts encoding GLUT1, GLUT3, GLUT4, GLUT8, GLUT10, and GLUT12 (Figure 1A and Additional file 1: Figure S1). A faint band that is the appropriate size to be GLUT5 was detected in a few but not all left ventricle cDNA samples in both C57BL/6J and FVB/NJ mice. We were not, however, able to subclone the GLUT5 amplicon derived from left ventricle cDNA into a pCR2.1-TOPO plasmid for use in the quantitative real time PCR described below, and therefore for the purposes of this study we consider the expression of GLUT5 in the murine left ventricle to be below detectable levels. We were unable to detect GLUT6 in the left ventricle of C57BL/6J mice. However, a band for GLUT6 in the left ventricle of FVB/NJ mice was observed. Transcripts for GLUT2 or GLUT9 in either C57BL/6J or FVB/NJ mice were not detected. PCRs were also run on cDNA isolated from liver, intestine, and brain (Figure 1B) to confirm the specificity of the primers for use in amplifying GLUT2 (liver), GLUT5 (intestine), GLUT6 (brain), and GLUT9 (liver). The identity of each GLUT amplicon was confirmed by sequencing.

### GLUT mRNA levels in murine left ventricles

Most of the current research investigating the role of glucose transporters in the myocardium has focused on the ‘classical’ Class I glucose transporters GLUT1 and GLUT4. Other facilitative GLUTs, including the more recently identified Class III GLUTs-8, -10, and −12, are also present in the heart. Several of these GLUTs are not yet well characterized, and it is possible that they also play a significant role in regulating glucose uptake in the heart. To understand better the extent to which these facilitative glucose transporters contribute to cardiac glucose utilization, we quantified the absolute levels of GLUT mRNA in left ventricles derived from C57BL/6J and FVB/NJ mice, two of the commonly used strains in genetic and surgical studies of cardiac hypertrophy. Tables 2 and 3 display the copy numbers of GLUT mRNA transcripts per nanogram of total RNA derived from murine left ventricles. We found that GLUT4, followed by GLUT1, is the predominant GLUT expressed in the murine left ventricle myocardia (Tables 2 and 3). mRNAs for GLUTs-3, -8, -10, and −12 are present in the left ventricle, but in considerably lower abundance. GLUT8 has the next highest mRNA level following GLUT4 and GLUT1. However, it is still not clear whether GLUT8 preferentially transports glucose or other hexoses *in vivo*, as well as if GLUT8’s primary role is at the cell surface or in an intracellular compartment, and thus its role in energy production in the heart is currently unclear. In FVB/NJ mice, GLUT12 is the fourth most abundant GLUT transcript in the LV after GLUT8. GLUT12 is currently hypothesized to act as a second insulin-responsive GLUT in addition to GLUT4. The functional role of GLUT12 in facilitating insulin-responsive glucose transport in this organ, however, has not been fully elucidated [16,20]. GLUT6 was expressed at almost undetectable levels in LVs from FVB/NJ mice.

**Table 2:** Glut mRNA quantification in murine left ventricle (copy number/ng total RNA)

	**C57BL/6J males (n = 8)**
GLUT1	982 ± 139
GLUT2	NA
GLUT3	62 ± 8
GLUT4	8878 ± 1532
GLUT5	NA
GLUT6	NA
GLUT8	360 ± 67
GLUT9	NA
GLUT10	73 ± 26
GLUT12	44 ± 5
ACTIN	15998 ± 2582

NA, Not applicable. Unable to detect transcript levels.

**Table 3:** Glut mRNA quantification in murine left ventricle (copy number/ng total RNA)

	**FVB/NJ males (n = 8)**
GLUT1	457 ± 75
GLUT2	NA
GLUT3	26 ± 2
GLUT4	3202 ± 447
GLUT5	NA
GLUT6	8 ± 2
GLUT8	268 ± 34
GLUT9	NA
GLUT10	18 ± 2
GLUT12	170 ± 22
ACTIN	9020 ± 1212

NA, Not applicable. Unable to detect transcript levels.

### GLUT transcript expression in GLUT4 null myocardium

**Figure 2:**
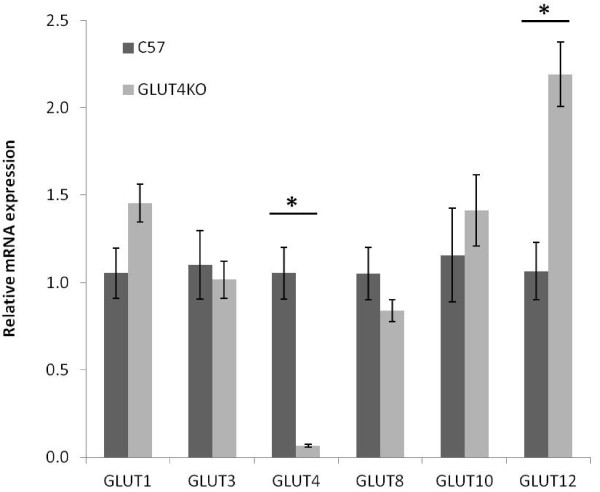
**Relative expression of GLUT transcripts in the left ventricle of the murine GLUT4 null model of cardiac hypertrophy.** Comparative real time PCR was performed to examine the relative mRNA expression of GLUT transcripts in left ventricles derived from six-month-old GLUT4 null and C57BL/6J mice. Real time PCR demonstrates an increase in GLUT12 mRNA expression in the left ventricles of GLUT4 null mice (n = 6 mice/group). *, *P* < 0.05.

GLUT4 is the predominant facilitative GLUT in insulin sensitive tissues including the heart, skeletal muscle, and adipose tissue. Quantitative data from this study shows that GLUT4 mRNA is roughly ten-fold more abundant than any other GLUT in the left ventricle. In spite of its major role in regulating glucose utilization in insulin-sensitive tissues, genetic ablation of GLUT4 does not result in overt diabetes but instead GLUT4 null mice exhibit a range of metabolic abnormalities including marked cardiac hypertrophy [21]. Even with the absence of the major glucose transporter, however, GLUT4 null hearts transport normal levels of glucose and synthesize normal levels of glycogen [22]. To determine whether other facilitative GLUTs are upregulated in the absence of GLUT4 in order to enable GLUT4 null hearts to maintain a high level of glucose metabolism, we examined the fold change in GLUT transcripts in RNA isolated from the left ventricles of six-month-old GLUT4 null males and age matched controls. As expected, our primers detected a 94% decrease in GLUT4 mRNA levels in the left ventricle of the GLUT4 null mouse. We did not observe a significant change in the transcript levels of GLUTs-1, -3, -8, or −10 in the left ventricles of GLUT4 null mice compared to wild type C57BL/6J controls (Figure 2). While GLUT1 levels were elevated in the left ventricles of GLUT4 null mice, this increase in transcript levels did not reach significance (p = 0.05). We did however observe a significant increase in the transcript levels of GLUT12 in the left ventricles of GLUT4 null mice.

### GLUT protein expression in GLUT4 null mice

**Figure 3:**
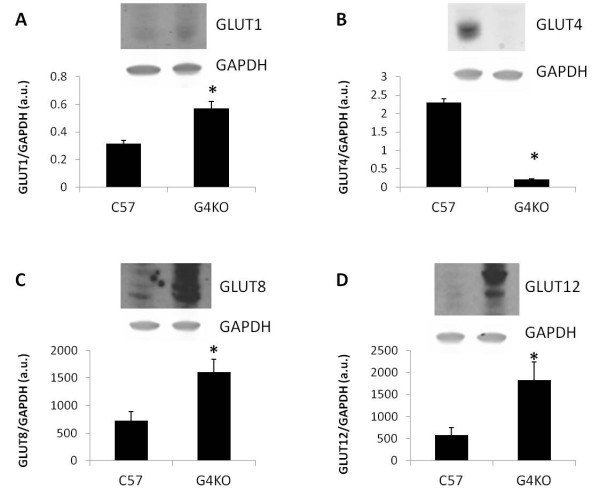
**Relative expression of GLUT protein in the left ventricle of GLUT4 null mice.** Western blotting was performed to examine the relative expression of GLUT1 (**A**), GLUT4 (**B**), GLUT8 (**C**), and GLUT12 (**D**) in the LVs of C57BL/6J and GLUT4 null mice. GLUT1, GLUT8, and GLUT12 protein expression is significantly elevated in the LVs of GLUT4 null mice compared to wild type controls (n = 3-4 mice/group). *, *P* < 0.05.

To determine whether changes in GLUT transcript levels correlate with changes in protein level in the GLUT4 null myocardium, we used western blotting to examine the protein expression of GLUT1, GLUT4, GLUT8, and GLUT12 in GLUT4 null mice and in wild type controls. Our data show that GLUT1, GLUT4, and GLUT8 are the most abundant GLUT transcripts in the left ventricle. Furthermore, GLUT12 mRNA levels are elevated in the left ventricle of GLUT4 null mice compared to wild type controls. As predicted, GLUT4 protein expression was dramatically diminished in the GLUT4 null myocardium (Figure 3). Our western blot data also show that the expression levels of GLUTs-1, -8, and −12 were significantly elevated in the GLUT4 null myocardium compared to wild type controls.

### GLUT transcript expression in genetic and surgical murine models of cardiomyopathy

**Figure 4:**
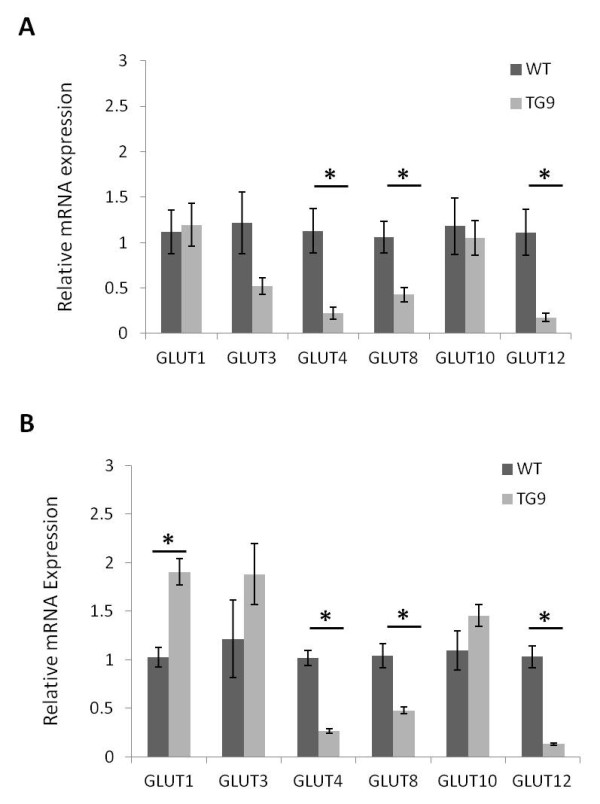
**Relative expression of GLUT transcripts in the left ventricle of the TG9 model of dilated cardiomyopathy.** Comparative real time PCR was performed to examine the relative mRNA expression of GLUT transcripts in left ventricles derived from A. 70-day-old and B. 85-day-old TG9 mice (TG9) and FVB/NJ controls (WT). Real time PCR shows that the expression levels of GLUT1 mRNA increase significantly in TG9 mice compared to age matched controls during heart failure progression. Conversely, the transcript levels for the insulin responsive glucose transporters GLUT4, GLUT8, and GLUT12 are significantly lower in heart from TG9 mice compared to FVB/NJ controls (n = 6–11 mice/group). *, *P* < 0.05.

To determine whether the myocardial expression levels of the more recently identified glucose transporters modulate in situations of chronic cardiac stress, we measured the fold change in GLUT transcripts in the left ventricle of a genetic murine model of dilated cardiomyopathy. TG9 mice develop echocardiographic evidence of left ventricular dilatation and systolic dysfunction by 8 weeks of age followed by a steady increase in chamber size and decrease of contractile function leading to decompensated heart failure and death between 11–13 and weeks [23]. Eighty-five day old male TG9 mice that were in advanced stages of heart failure were sacrificed and the left ventricle was harvested. RNA was isolated from the left ventricle and cDNA was synthesized as described above. Real time PCR showed that significant differences exist between GLUT transcript levels in the left ventricles of TG9 mice compared to age matched controls (Figure 4). At 70 days of age, which is just prior to the development of overt cardiac dysfunction [23], GLUT1 and GLUT10 transcript levels were comparable to wild-type control mice whereas message for the insulin-responsive GLUTs −4, -8 and −12 were significantly lower (Figure 4A). By 85 days of age, GLUT1 transcript levels were significantly elevated in the TG9 ventricles compared to age-matched wild-type controls (Figure 4B). GLUT3 transcript levels also increased, but differences in relative transcript levels between wild-type and TG9 mice did not reach significance (p = 0.08). Relative transcript levels of GLUTs −4, -8, and −12 remained lower in the TG9 left ventricle compared to wild type controls.

**Figure 5:**
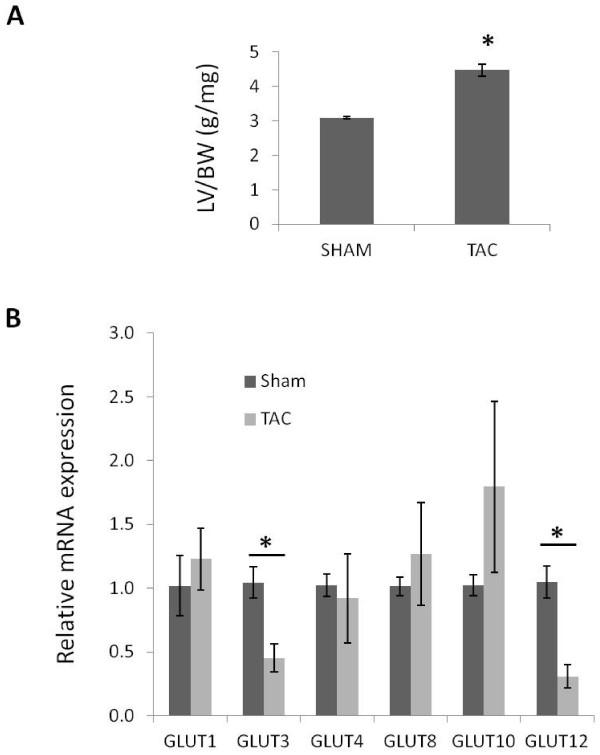
**Relative expression of GLUT transcripts in the left ventricle of a murine model of cardiac pressure overload.** Comparative real time PCR was performed to analyze the relative mRNA expression of GLUT transcripts in the left ventricles derived from C57BL/6J mice in a pressure-overload induced cardiac hypertrophy setting. **A**) An increased left ventricle to body weight ratio shows that mice receiving TAC were experiencing compensatory cardiac hypertrophy. **B**) Real time PCR shows that the expression levels of GLUT transcripts were lower in mice receiving aortic constriction compared to sham operated animals (n = 6-7 mice/group). *, *P* < 0.05.

To determine whether mRNA expression levels of the GLUTs similarly varies in a murine model of pathological hypertrophy induced by cardiac pressure overload, C57BL/6J male mice were subjected to aortic banding (or sham) surgeries. One week following surgery, RNA was isolated from left ventricles of surviving animals. As expected, the aortic banding surgery induced a significant degree of hypertrophy in the left ventricles (Figure 5A). Real time PCR showed that relative transcript levels were decreased for GLUTs −3 and 12 and unchanged for GLUTs −1, -4, -8 and −10 in mice one week following aortic banding (Figure 5B).

## Discussion

The uptake and metabolism of glucose significantly contributes to the production of myocardial ATP under normal conditions. Glucose assumes an even greater role in energy production in the heart during increased workload, ischemia, and pressure overload hypertrophy [1,2]. Because alterations in myocardial glucose utilization occur under conditions of cardiac injury or stress, better understanding of the factors that control the transport of glucose into the myocardium is vitally important for efforts to improve clinical outcomes in affected patients. The entry of glucose into the heart is mediated by members of the GLUT family of facilitative transporter proteins. While prior research has focused primarily on understanding the roles of the ubiquitous glucose transporter GLUT1 and the insulin-sensitive glucose transporter GLUT4 in regulating myocardial glucose uptake [21,22,24-26], less attention has been devoted to elucidating the roles of other GLUT isoforms in both the normal heart and in pathological states. To this end, we set out to define and characterize the presence and relative abundance of GLUT isoforms in the healthy adult murine left ventricle. Furthermore, we examined how the expression levels of each of these isoforms change under pathophysiological conditions.

### Functional implications of GLUT expression in the healthy heart

The current data show that mRNA encoding GLUTs-1, -3, -4, -8, -10, and −12 are present in the left ventricles of adult male FVB/NJ and C57BL/6J mice, two common strains of mice used for generating both genetic and surgical models of heart failure and stress. The presence of these isoforms in the murine left ventricle is in agreement with previous studies detecting these GLUTs in mammalian hearts [10-13,20,27]. GLUT6 was also detected in the left ventricles of FVB/NJ mice, which has not been previously reported. GLUT5 was detectable in some but not all of the left ventricles derived from adult male FVB/NJ and C57BL/6J mice. Due to the inability to subclone a GLUT5 amplicon derived from left ventricular cDNA into a pCR2.1-TOPO cloning vector to be used as a template for a standard curve, the presence of this transporter isoform in left ventricular myocardium could not be confirmed and relative expression levels could not be quantified. While trace levels of GLUT5 mRNA may be present in the myocardium, because the total RNA in this study was isolated from whole tissue rather than isolated cardiomyocytes, it is possible that the low level detection of GLUTs 5 and 6 may have been derived from small amounts of other cell types harvested along with the left ventricle.

Due to a diversity of detectable GLUTs in the heart, characterization of the relative abundance of each of these GLUTs in the heart is an important step towards understanding how each of these GLUTs may contribute towards maintaining myocardial glucose transport under healthy settings. Based on their relative transcript abundance in the left ventricles of both FVB/NJ and C57BL/6J mice, GLUT4, GLUT1, and GLUT8 likely serve important physiological roles in the healthy heart. The mRNA levels of GLUT3, GLUT6, GLUT10 and GLUT12 are comparatively less abundant. This may point to a more limited role for these isoforms in regulating myocardial glucose uptake under healthy circumstances. It is important to note, however, that the relative abundance of GLUT mRNAs in the LV may not directly correlate with the relative abundance of GLUT protein. Clearly, more research is needed to determine the precise roles that each of these transporters play in the heart and in other mammalian tissues. It is possible that several of the expressed GLUT isoforms have significant functions in this tissue that are distinct from their putative roles as glucose transporters.

The relative abundance of GLUT8 mRNA in the left ventricles of adult FVB/NJ and C57BL/6J mice suggests that this isoform may play a role in facilitating hexose transport across the membranes of myocardial cells. The functional significance of GLUT8-mediated transport in normal heart physiology is unclear. While GLUT8 has been reported to act as an insulin-responsive GLUT isoform in murine blastocysts [15], in other many mammalian cell types GLUT8 localizes primarily to lysosomal compartments and does not translocate to the cell surface in response to insulin. Additionally, unlike the genetic ablation of GLUT4, which results in severe cardiac hypertrophy in mice [21], the genetic ablation of GLUT8 does not influence cardiac size or morphology [28]. The GLUT8 null heart does however display an increased P-wave duration in electrocardiogram analysis, suggesting that this transporter isoform may influence atrial contraction [28]. Further investigation is warranted to determine whether GLUT8 directly influences myocardial glucose utilization or has a separate role in influencing normal cardiac physiology.

### Compensatory changes in myocardial GLUT expression following GLUT4 ablation

Our data show that GLUT4 mRNA in the left ventricle is more abundant than mRNA for every other GLUT isoform combined. Genetic ablation of GLUT4 in the heart has been shown to result in pronounced cardiac hypertrophy that is similar to hypertrophy that is induced by hypertension [21]. While the molecular mechanisms leading to hypertrophy in GLUT4 null mice are likely complex, hyperinsulinemia, hypertension and oxidative stress have been implicated as contributing factors [29,24]. Interestingly, despite the absence of the most abundant GLUT isoform in the heart, GLUT4 null mice continue to transport glucose into the heart at normal levels [22]. Previous studies have suggested that upregulation of the GLUT1 isoform is responsible for maintaining normal levels of glucose transport into the heart. Our study shows that in addition to GLUT1, the protein levels of the GLUT8 and GLUT12 isoforms and the transcript levels of the GLUT12 isoform are also elevated in the left ventricles of GLUT4 null mice. The observed discrepancies between GLUT gene and protein expression, which have been previously observed with GLUT1 and GLUT4, can be due to changes in translational regulation and/or protein stability [30,31].

The compensatory increase in the expression levels of other GLUT isoforms in the left ventricles of GLUT4 null mice complicates efforts to isolate the effect of GLUT4 ablation on cardiac function. Because the GLUT4 null heart transports glucose at normal levels, it can be inferred that alternate GLUT isoforms that are upregulated in the absence of GLUT4 serve a compensatory role in facilitating glucose uptake into the heart. These changes may influence glucose flux directly through increased levels of transporter protein within the plasma membrane or indirectly via alterations in cellular signaling pathways [32]. Similar alterations in GLUT isoform expression may also contribute to the observed dissociation between changes in GLUT4 expression and insulin sensitivity in other models such as exercise detraining [33].

Since the normal functions of the newer members of the GLUT family remain poorly characterized, it is possible that elevated GLUT8 and GLUT12 expression contributes to the hypertrophic phenotype observed in GLUT4 null hearts. The ability to disrupt glucose transport in an isoform-selective manner would provide a means to better clarify how each GLUT isoform participates in normal and pathological cardiac function in the absence of compensatory changes in GLUT expression. Our laboratory has previously demonstrated that the HIV protease inhibitor (PI) indinavir can acutely, selectively, and reversibly inhibit GLUT4 activity *in vitro* and *in vivo*[34-36]. Ongoing efforts to identify the structural basis for PI binding to GLUTs will aide in the development of novel pharmacological agents that specifically inhibit other GLUT isoforms. This development would enrich our ability to explore how each GLUT isoform affects energy homeostasis in specific mammalian tissues.

Previous studies have demonstrated that isolated soleus muscle of female GLUT4 null mice displays augmented glucose uptake in response to insulin stimulation [37]. This demonstrates that an additional isoform is able to compensate at least partially for the genetic ablation of GLUT4 by acting as an insulin-responsive GLUT. At the time that this observation was made, several GLUTs including GLUT12 had not yet been identified. The increased expression of GLUT12 in GLUT4 null mice provides evidence that GLUT12 mediates the observed increased glucose uptake. Recent studies have demonstrated that GLUT12 traffics to the cell surface of skeletal muscle in response to insulin-stimulation [38] and that GLUT12 overexpression in mice improves insulin sensitivity [16]. The increase in GLUT12 mRNA expression in the GLUT4 null LVs suggests that GLUT12 may be playing a role as a second insulin responsive GLUT in the myocardium. It is still unclear whether GLUT12 translocates to the cell surface in response to contractile stimulation. Because the expression level of GLUT4 is normally significantly greater than GLUT12, it is unclear how much GLUT12 contributes to maintaining myocardial glucose homeostasis in the healthy heart.

### Changes in myocardial GLUT expression under pathophysiologic conditions

Although mRNAs for GLUTs-3, -8, -10, and −12 are present in the LV at comparatively lower levels than GLUTs-1 and −4, it is possible that these isoforms play a greater role in regulating myocardial glucose uptake under pathological circumstances. We therefore examined the expression levels of each GLUT isoform in TG9 mice in advanced stages of dilated cardiomyopathy, and in murine surgical model of pressure overload hypertrophy. We found that the transcripts for GLUT1 and GLUT3 increased as heart failure developed in TG9 mice while transcripts for GLUT4, GLUT8, and GLUT12 were all decreased. The upregulation of both GLUT1 and GLUT3 in TG9 mice is consistent with prior reports of altered gene transcription during heart failure [39]. Both GLUT1 as well as GLUT3 are commonly found in high abundance in fetal tissues [40] and GLUT1 is the major GLUT expressed in the fetal heart [7]. The relative changes in mRNA transcript levels observed in the aortic banding model, however, demonstrate that pathologic cardiac hypertrophy is not necessarily accompanied by an increase in GLUT1 mRNA expression.

The decreased expression of GLUT12 in both the TG9 and aortic banding model in our study contrasts with a previous report of increased GLUT12 mRNA levels in a canine tachypacing heart failure model [20]. It is possible that this discrepancy reflects differences between species and specific molecular mechanisms for heart failure. This underscores the need for caution against overgeneralization of the findings from unique heart failure models. Clearly, continued efforts to more extensively identify and characterize the complex cellular changes that occur during heart failure development will allow greater insight into the mechanisms and functional consequences of altered myocardial GLUT expression.

## Conclusions

Our data show that at least six members of the facilitative GLUT family are detectable in murine left ventricular tissue. Significant changes occur in mRNA message levels in mice with heart disease induced by different mechanisms. It remains to be determined whether these changes are adaptive or maladaptive. Continuing efforts to identify and characterize the functional significance of each of these isoforms in the myocardium will further enhance our understanding of myocardial energy dynamics under both healthy conditions and pathological circumstances. This work will serve as a basis for future efforts to investigate the individual roles of these newly identified GLUT isoforms in regulating myocardial energy dynamics.

## Abbreviations

PCR, Polymerase chain reaction; GLUT, Facilitative glucose transport protein; HMIT, (H+) myo-inositol transporter; LV, Left ventricle; TAC, Transverse aortic constriction; GAPDH, Glyceraldehyde 3-phosphate dehydrogenase; HPRT, Hypoxanthine-guanine phosphoribosyl-transferase; PBS, Phosphate buffered saline.

## Competing interests

The authors declare that they have no competing interests.

## Authors’ contributions

LAF conceived the study, assisted in the design, execution and interpretation of experiments, and drafted the manuscript. MA, MP and AK assisted in performing experiments. PH provided guidance in experimental design, performed data analysis and interpretation, and assisted in writing and editing the manuscript. All authors read and approved the final manuscript.

## Supplementary Material

Additional file 1 **Figure S1.Detection of GLUT mRNAs in the left ventricle of FVB/NJ mice. A**) PCR was employed to identify each known murine member of the facilitative glucose transporter family in the left ventricle of 10-week-old male FVB/NJ mice. GLUTs-1, -3, -4, -6, -8, -10, and −12 were amplified using cDNA derived from the left ventricular myocardium. The identity of each GLUT amplicon was confirmed by sequencing. A faint band for GLUT5 was detected in some but not all of the left ventricles analyzed. GLUTs-2 and −9 were not present in the left ventricle of male C57BL/6J mice. Representative images are shown (n = 3). **B**) The specificity of the primers used to amplify GLUTs-2 and −9 were confirmed using cDNA derived from liver and intestine, respectively. The specificity of the primers for each GLUT amplicon was confirmed by sequencing. Click here for file

## References

[B1] Kolwicz SC Tian R Metabolic therapy at the crossroad: how to optimize myocardial substrate utilization? Trends Cardiovasc Med 2009 19 6 201 207 10.1016/j.tcm.2009.12.005 20211436 PMC2836268

[B2] Stanley WC Recchia FA Lopaschuk GD Myocardial substrate metabolism in the normal and failing heart Physiol Rev 2005 85 3 1093 1129 10.1152/physrev.00006.2004 15987803

[B3] Ingwall JS Weiss RG Is the failing heart energy starved? On using chemical energy to support cardiac function Circ Res 2004 95 2 135 145 10.1161/01.RES.0000137170.41939.d9 15271865

[B4] Wood IS Trayhurn P Glucose transporters (GLUT and SGLT): expanded families of sugar transport proteins Br J Nutr 2003 89 1 3 9 10.1079/BJN2002763 12568659

[B5] Thorens B Mueckler M Glucose transporters in the 21st Century Am J Physiol Endocrinol Metab 2010 298 2 E141 E145 10.1152/ajpendo.00712.2009 20009031 PMC2822486

[B6] Studelska DR Campbell C Pang S Rodnick KJ James DE Developmental expression of insulin-regulatable glucose transporter GLUT-4 Am J Physiol 1992 263 1 Pt 1 E102 E106 1636686 10.1152/ajpendo.1992.263.1.E102

[B7] Smoak IW Branch S Glut-1 expression and its response to hypoglycemia in the embryonic mouse heart Anat Embryol (Berl) 2000 201 5 327 333 10.1007/s004290050321 10839628

[B8] Brosius FC Schwaiger M Bartlett J Sun D Nguyen N Liu Y Persistent myocardial ischemia increases GLUT1 glucose transporter expression in both ischemic and non-ischemic heart regions J Mol Cell Cardiol 1997 29 6 1675 1685 10.1006/jmcc.1997.0405 9220353

[B9] Razeghi P Young ME Alcorn JL Moravec CS Frazier OH Taegtmeyer H Metabolic gene expression in fetal and failing human heart Circulation 2001 104 24 2923 2931 10.1161/hc4901.100526 11739307

[B10] Abel ED Glucose transport in the heart Front Biosci 2004 9 201 215 10.2741/1216 14766360

[B11] Grover-McKay M Walsh SA Thompson SA Glucose transporter 3 (GLUT3) protein is present in human myocardium Biochim Biophys Acta 1999 1416 1–2 145 154 9889355 10.1016/s0005-2736(98)00216-8

[B12] Doege H Schurmann A Bahrenberg G Brauers A Joost HG GLUT8, a novel member of the sugar transport facilitator family with glucose transport activity J Biol Chem 2000 275 21 16275 16280 10.1074/jbc.275.21.16275 10821868

[B13] Dawson PA Mychaleckyj JC Fossey SC Mihic SJ Craddock AL Bowden DW Sequence and functional analysis of GLUT10: a glucose transporter in the Type 2 diabetes-linked region of chromosome 20q12–13.1 Mol Genet Metab 2001 74 1–2 186 199 11592815 10.1006/mgme.2001.3212

[B14] Macheda ML Kelly DJ Best JD Rogers S Expression during rat fetal development of GLUT12–a member of the class III hexose transporter family Anat Embryol Berl 2002 205 5–6 441 452 12382147 10.1007/s00429-002-0263-8

[B15] Carayannopoulos MO Chi MM Cui Y Pingsterhaus JM McKnight RA Mueckler M Devaskar SU Moley KH GLUT8 is a glucose transporter responsible for insulin-stimulated glucose uptake in the blastocyst Proc Natl Acad Sci U S A 2000 97 13 7313 7318 10.1073/pnas.97.13.7313 10860996 PMC16542

[B16] Purcell SH Aerni-Flessner LB Willcockson AR Diggs-Andrews KA Fisher SJ Moley KH Improved insulin sensitivity by GLUT12 overexpression in mice Diabetes 2011 60 5 1478 1482 10.2337/db11-0033 21441439 PMC3292321

[B17] Rogers JH Tamirisa P Kovacs A Weinheimer C Courtois M Blumer KJ Kelly DP Muslin AJ RGS4 causes increased mortality and reduced cardiac hypertrophy in response to pressure overload J Clin Invest 1999 104 5 567 576 10.1172/JCI6713 10487771 PMC408537

[B18] Frolova AI Moley KH Quantitative analysis of glucose transporter mRNAs in endometrial stromal cells reveals critical role of GLUT1 in uterine receptivity Endocrinology 2011 152 5 2123 2128 10.1210/en.2010-1266 21343253 PMC3075937

[B19] Feldman A Ray P Silan C Mercer J Minobe W Bristow M Selective gene expression in failing human heart. Quantification of steady-state levels of messenger RNA in endomyocardial biopsies using the polymerase chain reaction Circulation 1991 83 6 1866 1872 10.1161/01.CIR.83.6.1866 2040039

[B20] Ware B Bevier M Nishijima Y Rogers S Carnes CA Lacombe VA Chronic heart failure selectively induces regional heterogeneity of insulin-responsive glucose transporters Am J Physiol Regul Integr Comp Physiol 2011 301 5 R1300 R1306 10.1152/ajpregu.00822.2010 21849635 PMC3213947

[B21] Katz EB Stenbit AE Hatton K DePinho R Charron MJ Cardiac and adipose tissue abnormalities but not diabetes in mice deficient in GLUT4 Nature 1995 377 6545 151 155 10.1038/377151a0 7675081

[B22] Stenbit AE Katz EB Chatham JC Geenen DL Factor SM Weiss RG Tsao TS Malhotra A Chacko VP Ocampo C Preservation of glucose metabolism in hypertrophic GLUT4-null hearts Am J Physiol Heart Circ Physiol 2000 279 1 H313 H318 10899071 10.1152/ajpheart.2000.279.1.H313

[B23] Buerger A Rozhitskaya O Sherwood MC Dorfman AL Bisping E Abel ED Pu WT Izumo S Jay PY Dilated cardiomyopathy resulting from high-level myocardial expression of Cre-recombinase J Card Fail 2006 12 5 392 398 10.1016/j.cardfail.2006.03.002 16762803

[B24] Abel ED Kaulbach HC Tian R Hopkins JC Duffy J Doetschman T Minnemann T Boers ME Hadro E Oberste-Berghaus C Cardiac hypertrophy with preserved contractile function after selective deletion of GLUT4 from the heart J Clin Invest 1999 104 12 1703 1714 10.1172/JCI7605 10606624 PMC409881

[B25] Liao R Jain M Cui L D'Agostino J Aiello F Luptak I Ngoy S Mortensen RM Tian R Cardiac-specific overexpression of GLUT1 prevents the development of heart failure attributable to pressure overload in mice Circulation 2002 106 16 2125 2131 10.1161/01.CIR.0000034049.61181.F3 12379584

[B26] Luptak I Yan J Cui L Jain M Liao R Tian R Long-term effects of increased glucose entry on mouse hearts during normal aging and ischemic stress Circulation 2007 116 8 901 909 10.1161/CIRCULATIONAHA.107.691253 17679614

[B27] Machado UF Shimizu I Saito M Reduced content and preserved translocation of glucose transporter (GLUT 4) in white adipose tissue of obese mice Physiol Behav 1994 55 4 621 625 10.1016/0031-9384(94)90035-3 8190786

[B28] Membrez M Hummler E Beermann F Haefliger JA Savioz R Pedrazzini T Thorens B GLUT8 is dispensable for embryonic development but influences hippocampal neurogenesis and heart function Mol Cell Biol 2006 26 11 4268 4276 10.1128/MCB.00081-06 16705176 PMC1489108

[B29] Li Y Wende AR Nunthakungwan O Huang Y Hu E Jin H Boudina S Abel ED Jalili T Cytosolic, but not mitochondrial, oxidative stress is a likely contributor to cardiac hypertrophy resulting from cardiac specific GLUT4 deletion in mice FEBS J 2012 279 4 599 611 10.1111/j.1742-4658.2011.08450.x 22221582 PMC3267000

[B30] Santalucía T Camps M Castelló A Muñoz P Nuel A Testar X Palacin M Zorzano A Developmental regulation of GLUT-1 (erythroid/Hep G2) and GLUT-4 (muscle/fat) glucose transporter expression in rat heart, skeletal muscle, and brown adipose tissue Endocrinology 1992 130 2 837 846 10.1210/en.130.2.837 1370797

[B31] Bourey RE Koranyi L James DE Mueckler M Permutt MA Effects of altered glucose homeostasis on glucose transporter expression in skeletal muscle of the rat J Clin Invest 1990 86 2 542 547 10.1172/JCI114742 2384600 PMC296758

[B32] Buller CL Heilig CW Brosius FC GLUT1 enhances mTOR activity independently of TSC2 and AMPK Am J Physiol Renal Physiol 2011 301 3 F588 F596 10.1152/ajprenal.00472.2010 21613414 PMC3174561

[B33] Lehnen AM Leguisamo NM Pinto GH Markoski MM De Angelis K Machado UF Schaan B The beneficial effects of exercise in rodents are preserved after detraining: a phenomenon unrelated to GLUT4 expression Cardiovasc Diabetol 2010 9 67 10.1186/1475-2840-9-67 21029425 PMC2984487

[B34] Murata H Hruz PW Mueckler M The mechanism of insulin resistance caused by HIV protease inhibitor therapy J Biol Chem 2000 275 27 20251 20254 10.1074/jbc.C000228200 10806189

[B35] Murata H Hruz PW Mueckler M Indinavir inhibits the glucose transporter isoform Glut4 at physiologic concentrations AIDS 2002 16 6 859 863 10.1097/00002030-200204120-00005 11919487

[B36] Hresko RC Hruz PW HIV Protease Inhibitors Act as Competitive Inhibitors of the Cytoplasmic Glucose Binding Site of GLUTs with Differing Affinities for GLUT1 and GLUT4 PLoS One 2011 6 9 e25237 10.1371/journal.pone.0025237 21966466 PMC3179492

[B37] Stenbit AE Burcelin R Katz EB Tsao TS Gautier N Charron MJ Le Marchand-Brustel Y Diverse effects of Glut 4 ablation on glucose uptake and glycogen synthesis in red and white skeletal muscle J Clin Invest 1996 98 3 629 634 10.1172/JCI118833 8698853 PMC507471

[B38] Stuart CA Howell ME Zhang Y Yin D Insulin-stimulated translocation of glucose transporter (GLUT) 12 parallels that of GLUT4 in normal muscle J Clin Endocrinol Metab 2009 94 9 3535 3542 10.1210/jc.2009-0162 19549745 PMC2741719

[B39] Rosenblatt-Velin N Montessuit C Papageorgiou I Terrand J Lerch R Postinfarction heart failure in rats is associated with upregulation of GLUT-1 and downregulation of genes of fatty acid metabolism Cardiovasc Res 2001 52 3 407 416 10.1016/S0008-6363(01)00393-5 11738057

[B40] Matsumoto K Akazawa S Ishibashi M Trocino RA Matsuo H Yamasaki H Yamaguchi Y Nagamatsu S Nagataki S Abundant expression of GLUT1 and GLUT3 in rat embryo during the early organogenesis period Biochem Biophys Res Commun 1995 209 1 95 102 10.1006/bbrc.1995.1475 7726869

