# Supplementary figures and images for: GLUT4, GLUT1, and GLUT8 are the dominant GLUT transcripts expressed in the murine left ventricle
Source: Cardiovasc Diabetol. 2012 Jun 8;11:63. doi: 10.1186/1475-2840-11-63 (PMC3416696; doi:10.1186/1475-2840-11-63)

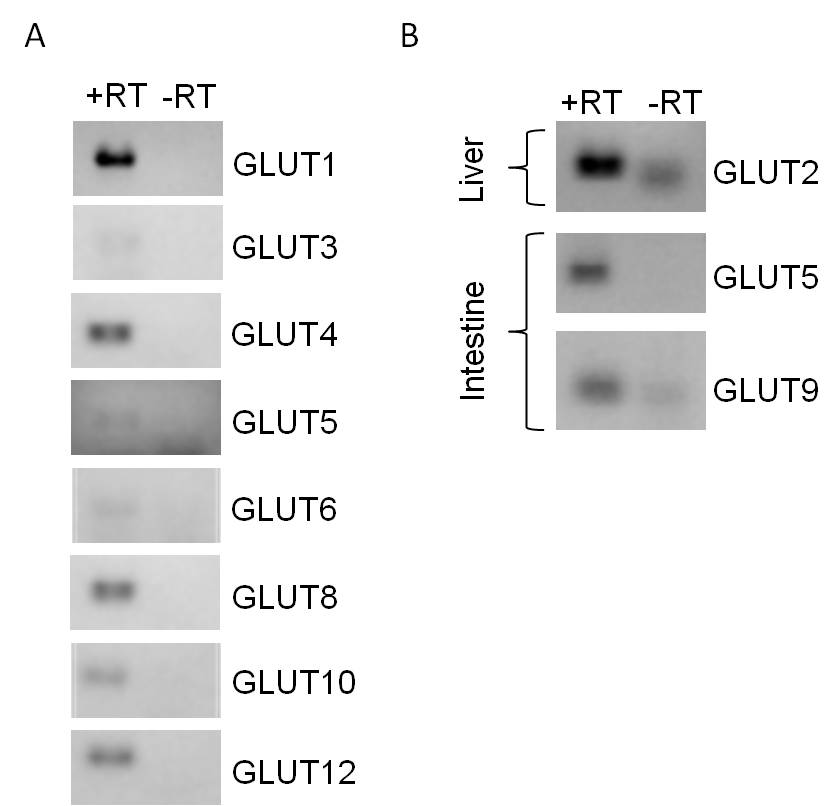

Supplement: Additional file 1 — Figure S1.Detection of GLUT mRNAs in the left ventricle of FVB/NJ mice. A) PCR was employed to identify each known murine member of the facilitative glucose transporter family in the left ventricle of 10-week-old male FVB/NJ mice. GLUTs-1, -3, -4, -6, -8, -10, and −12 were amplified using cDNA derived from the left ventricular myocardium. The identity of each GLUT amplicon was confirmed by sequencing. A faint band for GLUT5 was detected in some but not all of the left ventricles analyzed. GLUTs-2 and −9 were not present in the left ventricle of male C57BL/6J mice. Representative images are shown (n = 3). B) The specificity of the primers used to amplify GLUTs-2 and −9 were confirmed using cDNA derived from liver and intestine, respectively. The specificity of the primers for each GLUT amplicon was confirmed by sequencing. [file 1475-2840-11-63-S1.jpeg]
